# Comparison of VividTrac®, Airtraq®, King Vision®, Macintosh Laryngoscope and a Custom-Made Videolaryngoscope for difficult and normal airways in mannequins by novices

**DOI:** 10.1186/s12871-017-0362-y

**Published:** 2017-05-26

**Authors:** Szilárd Rendeki, Dóra Keresztes, Gábor Woth, Ákos Mérei, Martin Rozanovic, Mátyás Rendeki, József Farkas, Diána Mühl, Bálint Nagy

**Affiliations:** 1 Department of Anesthesiology and Intensive Therapy, Medical School, University of Pécs, Ifjúság Str. 13, HU-7624 Pécs, Hungary; 2 Medical Skills Lab, Medical School, University of Pécs, Szigeti Str. 12, HU-7624 Pécs, Hungary; 3 Department of Operational Medicine, Medical School, University of Pécs, Szigeti Str. 12, HU-7624 Pécs, Hungary; 4 Medical School, University of Pécs, Szigeti Str. 12, HU-7624 Pécs, Hungary; 5 Department of Anatomy, Medical School, University of Pécs, Szigeti Str. 12, HU-7624 Pécs, Hungary

**Keywords:** Airtraq®, Airway management, Endoscope, Improvised device, Intubation, King Vision®, Macintosh laryngoscope, Manikin, MILS, Novice user, User satisfaction, Smartphone, Videolaryngoscope, VividTrac®

## Abstract

**Background:**

Direct laryngoscopy remains the gold standard for endotracheal intubation and is preferred by experienced operators. However, an increasing number of reports currently support videolaryngoscopy, particularly for novice users. The widespread use of videolaryngoscopy may be limited due to financial limitations, especially in low-income countries. Therefore, affordable single-use scopes are now becoming increasingly popular. We sought to compare these new scopes with direct laryngoscopes and the previously tested videolaryngoscopes in mannequins by novices.

**Methods:**

Fifty medical students were recruited to serve as novice users. Following brief, standardized training, students were asked to execute endotracheal intubation with each of the devices, including the Airtraq®, a custom-made videolaryngoscope, the King Vision®, the Macintosh laryngoscope and the VividTrac®, on an airway trainer (Laerdal Airway Management Trainer®) in normal and difficult airway scenarios. We evaluated the time to and the proportion of successful intubation, the best view of the glottis, esophageal intubation, dental trauma and user satisfaction.

**Results:**

We observed no differences in esophageal intubation. However, intubation-related times, the view of the glottis and operator satisfaction were significantly better throughout the study with the commercial videolaryngoscopes. In comparison, the custom-made videolaryngoscope performance proved to be similar to that of the Macintosh laryngoscope. The VividTrac® performance was similar (*P* > 0.05) or significantly better than that of the King Vision® in both scenarios.

**Conclusions:**

Based upon our results, the Airtraq®, King Vision® and VividTrac® were superior to the Macintosh laryngscope in both normal and difficult airway scencarios for novice users. In particular, our study is the first to report that the VividTrac® shows promise for further clinical evaluation.

## Background

In clinical practice, orotracheal intubation with direct laryngoscopy (DL) is the preferred means of establishing a definitive airway in the majority of cases. Although DL is a well-known and reliable technique in the hands of an experienced operator, airway management is an urgent task that may need to be carried out regardless of specialty background to prevent impending disastrous complications, such as hypoxia and aspiration [1, 2]. Videolaryngoscopy (VL) might be beneficial compared to DL for novices, although the role of VL in airway management remains controversial [3, 4].

VL has evolved in the past 10 years, and more than ten different operational devices are currently available on the market. Although patients may benefit from the availability of VL, especially in difficult airway situations, the clinical availability of VL remains limited, especially in low- and middle-income countries [5–7]. Therefore, ongoing development now includes reducing VL costs, such as utilizing smartphones to display, store and share real-time videos. Low-cost custom-made devices are also available and have already been partially tested [6, 8].

The VividTrac® (VT, Vivid Medical, Palo Alto, USA) has been on the market since 2013 and is generally viewed as an inexpensive (<100 $), single-use VL. Currently, no data are available relative to VT clinical performance. However, the technical parameters of this device have already been evaluated with promising results [9].

A custom-made improvised VL (ID) was first reported in 2014 by Karippacheril et al. [8]. This device was originally assembled using a Universal Serial Bus (USB) endoscopic camera, a conventional Macintosh laryngoscope blade, and a PC. Reportedly, its performance has been deemed safe and reliable [8].

Therefore, our primary objective was to compare the VT, ID and commercially available VLs (Airtraq®, King Vision®) to DL in a standardized setting with novice users. Our secondary objective was to evaluate the functionality of the aforementioned devices, including operator satisfaction.

## Methods

### Ethics and sample size calculation

Prior to this study, permission was first obtained from the Institutional Scientific and Human Research Ethics Committee of the University of Pécs (5825/2016). The investigation was carried out at the Medical Skills Lab of the Medical School, University of Pécs, Hungary. Based on previous similar studies, we performed a sample size estimation prior to recruitment, using α = 0.05 and β = 0.1. We determined that a minimum of 48 participants was required for pair-wise comparisons of our samples [10, 11].

### Devices

**Fig. 1:**
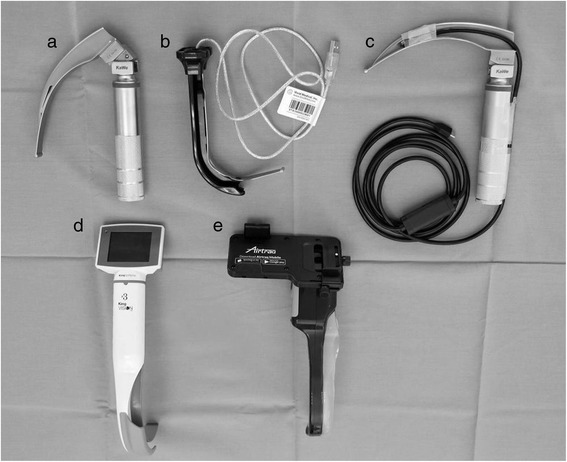
Evaluated laryngoscopes. **a** Direct laryngoscope (Macintosh); **b** VividTrac®; **c** Custom-made, improvised laryngoscope; **d** King Vision®; **e** Airtraq®

The following devices were included in this study: (a) DL with a size 3 blade (KaWe®, Asperg, Germany); (b) VT with an adult channeled blade (Vivid Medical, Palo Alto, USA); (c) ID assembled as previously reported [8]; (d) King Vision® (KV) with a size 3 channeled blade (Ambu, Copenhagen, Denmark); and (e) Airtraq® (AT) with a size 3 channeled blade (Prodol, Vizcaya, Spain) (Fig. 1).

For the ID and VT, we used a PC to display real-time videos during the study. For the AT, we attached the original, universal, smartphone adapter (Prodol, Vizcaya, Spain) and a smartphone to the scope for the same purpose. VividVision® and Airtraq Mobile® software was used for the VT and AT, respectively.

### Training

Two airway management scenarios were defined. In “Scenario A”, full head reclination was allowed, but in “Scenario B”, the cervical spine was fully immobilized manually as recommended by the Advanced Trauma Life Support algorithm [12]. Each participant received 15 min of standardized training on each device and in each study setting. Optimization maneuvers, the use of stylets and an estimation of the Percent of Glottic Opening (POGO) score were also explained and practiced under the supervision of experienced investigators [13]. The importance and the mechanism of dental injury were also highlighted. Each endotracheal intubation was performed with a standard 7.5-mm internal diameter, cuffed, plastic endotracheal tube (Mallinckrodt®, Covidien, Dublin, Ireland). Demonstrations, training and evaluations were all performed on the Laerdal® Airway Management Trainer (Laerdal®, Stavanger, Norway) [2, 10, 11, 14].

### Evaluation

Participants were asked to complete endotracheal intubations with all devices in both scenarios in a random order. The primary outcome was successful endotracheal intubation. Secondary outcomes included the time to successful endotracheal intubation, the time to best glottis view, tube insertion time, the best POGO achieved, the number of intubation attempts, the occurrence of esophageal intubations, the occurrence of dental trauma and the need for optimization maneuvers. The time elapsed from the tool blade passing the interdental line until the best POGO (marked as manipulation initiation with the endotracheal tube) was considered the laryngoscopy time (LT). The time to successful tracheal intubation was noted as the intubation time (IT), and the difference between IT and LT was registered as the tube insertion time (TIT). The following attempts were considered failed attempts: attempts that required more than 120 s, esophageal intubation (recognized by the participant), or the device was removed from the oral cavity during the attempt. The following intubations were considered failed: more than 3 unsuccessful attempts, esophageal intubation (not recognized by the participant), or the participant considered further attempts futile. Stylet use and POGO scores were reported by the participants (direct laryngoscopy) or the investigators (videolaryngoscopy) and were also noted.

Following the completion of a scenario, the students were asked to grade each device based on the ease of technical use (1 = easy, and 5 = difficult), the ease of physical use (1 = easy, and 5 = difficult) and the willingness to reuse (1 = would never use again, and 5 = would like to use) in the relevant scenario, but they were discouraged from overall ranking of the devices.

### Statistical analysis

The analyses were conducted by Statistical Package for the Social Sciences (SPSS) Statistics software, version 22.0 (IBM Corporation, Armonk, NY, USA). Continuous and ordinal data are presented as the median and interquartile range (IQR), and the categorical data are presented as raw numbers and as frequencies. Non-parametric tests were used because the data distribution was not normal based on Kolmogorov-Smirnov and Shapiro-Wilk tests. The Kruskal-Wallis one-way analysis of variance (ANOVA) with post-hoc Dunn’s test was used to assess pair-wise differences between the devices for the following variables: laryngoscopy time (LT), tube insertion time (TIT), intubation time (IT), POGO score, ease of technical use, ease of physical use and willingness to reuse. Chi-square tests were used to evaluate differences between the devices for the rate of successful tracheal intubation, esophageal intubation, dental injury and bougie and stylet usage. Values of *P* < 0.05 were considered significant.

## Results

Fifty voluntary medical students without prior experience in advanced airway management were recruited. All students provided written informed consent prior to participation.

### Scenario A

The acquired data are shown in detail in Table 1. No significant difference was observed in the tracheal intubation success rate between the devices throughout this scenario. The overall longest IT was associated with the ID, and commercial VLs were faster than DL. Participants achieved better POGO scores with all VLs than with the DL. In the comparison of the VLs relative to POGO score, the ID was found to be inferior (*P* < 0.05), but the VT proved to be far superior to the DL and ID (*P* < 0.05). All commercial VLs received better ease of use scores than the DL and ID (*P* < 0.05). The grades related to the willingness to reuse were significantly better for KV and VT than for the DL.

**Table 1:** Results of “Scenario A”

Scenario A	DL (*n* = 50)	ID (*n* = 50)	KV (*n* = 50)	AT (*n* = 50)	VT (*n* = 50)
Number of attempts (n, 1/2/3)	49/1/0	50/0/0	50/0/0	48/2/0	50/0/0
Laryngoscopy time (s)	9.46 [6.95–12.87]^†¶^	11.7 [9.11–15.1]^†§¶^	6.91 [5.59–10.1]^*#^	8.01 [6.21–10.2]^#¶^	5.87 [4.77–7.97]^*#§^
Tube insertion time (s)	4.98 [4.01–7.02]^§^	6.70 [5.49–9.47]^†§¶^	4.61 [2.81–6.27] ^#§^	3.04 [2.36–4.16]^*#†^	3.90 [2.20–7.07]^#^
Intubation time (s)	15.3 [11.92–20.5]^§¶^	19.7 [15.2–25.8]^†§¶^	12.7 [9.35–17.8]^#^	11.2 [8.7–14.04]^*#^	10.5 [7.55–14.3]^*#^
POGO (%)	80 [60, 80]^†§¶^	77.5 [60–90]^†§¶^	90 [83.75–95]^*#^	90 [80–95]^*#^	95 [90–100]^*#^
Ease of technical use (1–5)	3 [2–4]^†§¶^	3 [2–4]^†§¶^	1 [1–2]^*#^	2 [1–3]^*#†¶^	1 [1–2]^*#§^
Ease of physical use (1–5)	4 [3–4]^†§¶^	3 [3–4]^†§¶^	1 [1–2]^*#^	2 [1–2]^*#¶^	1 [1–2]^*#§^
Willingness of reuse (1–5)	4 [3–5]^†^	3 [2–4]^†¶^	5 [4–5]^*#§^	4 [3–5]^†¶^	5 [3–5]^#§^
Use of bougie (n)	0^#^	4^*†§¶^	0^#^	0^#^	0^#^
Use of stylet (n)	1	3	0	0	0
Dental injury (n)	26^#†§¶^	16^*†§¶^	7^*#^	5^*#¶^	10^*#§^
Esophageal intubation (n)	0	0	0	0	0

Data are reported as the median [IQR] or as numbers (n)

*AT* Airtraq®, *DL* Direct laryngoscope (Macintosh), *ID* Custom-made, improvised laryngoscope, *KV* King Vision®, *POGO* Percent of Glottic Opening, *VT* VividTrac®

^*^Significant difference (*P* < 0.05) compared to DL; ^#^Significant difference (*P* < 0.05) compared to ID; ^†^Significant difference (*P* < 0.05) compared to KV; ^§^Significant difference (*P* < 0.05) compared to AT; ^¶^Significant difference (*P* < 0.05) compared to VT

### Scenario B

Data are shown in detail in Table 2. Compared to the DL, little or no difference was observed in the first-time success rate of intubation using VLs (*P* > 0.05). However, within the VL group, the VT was found to be superior to the KV (*P* < 0.05) regarding first-time success rate. The ID revealed the slowest IT in the VL group (*P* < 0.05). The fastest devices for IT were the VT and the AT (*P* < 0.05). All VLs, excluding the ID, performed significantly better for POGO than the DL. Comparing the VLs, the highest POGO scores were achieved by the KV and AT.

**Table 2:** Results of “Scenario B”

Scenario B	DL (*n* = 50)	ID (*n* = 50)	KV (*n* = 50)	AT (*n* = 50)	VT (*n* = 50)
Number of attempts (n, 1/2/3)	48/1/1	47/2/1	46/4/0^¶^	47/3/0	50/0/0^†^
Laryngoscopy time (s)	12.16 [9.05–14.4]^#¶^	16.2 [11.7–23.4]^*†§¶^	10.86 [7.66–13.0]^#^	9.13 [7.37–11.7]^#^	8.99 [7.22–11.3]^*#^
Tube insertion time (s)	6.52 [4.33–12.97]^†§¶^	7.04 [5.45–15.04]^†§¶^	3.31 [2.05–11.68]^*#^	2.60 [1.90–4.87]^*#^	3.17 [2.13–4.87]^*#^
Intubation time (s)	19.0 [14.97–26.1]^§¶^	23.4 [19.0–35.5]^†§¶^	15.72 [11.5–23.1]^#^	12.8 [9.62–16.5]^*#^	12.7 [10.0–15.8]^*#^
POGO (%)	40 [20–60]^†§¶^	45 [25–55]^†§¶^	75 [60–85]^*#^	75 [60–85]^*#^	62.5 [50–90]^*#^
Ease of technical use (1–5)	4 [3–4]^†§¶^	4 [3–4]^†§¶^	2 [1–3]^*#^	2 [2–3]^*#^	2 [1–2]^*#^
Ease of physical use (1–5)	4 [3–5]^†§¶^	4 [3–5]^†§¶^	2 [1–3]^*#^	2 [2–3]^*#^	2 [1–2]^*#^
Willingness of reuse (1–5)	3 [2–4]^†¶^	3 [2–3]^†¶^	5 [4–5]^*#§^	3 [3–4]^†¶^	5 [4–5]^*#§^
Use of bougie (n)	10^†§¶^	9^†§¶^	0^*#^	0^*#^	0^*#^
Use of stylet (n)	5^#†§¶^	11^*†§¶^	0^*#^	0^*#^	0^*#^
Dental injury (n)	32^#§^	41^*†¶^	35^#§^	39^*†¶^	35^#§^
Esophageal intubation (n)	1	0	0	0	0

Data are reported as the median [IQR] or as numbers (n)

*AT* Airtraq®, *DL* Direct laryngoscope (Macintosh), *ID* Custom-made, improvised laryngoscope, *KV* King Vision®, *POGO* Percent of Glottic Opening, *VT* VividTrac®

^*^Significant difference (*P* < 0.05) compared to DL; ^#^Significant difference (*P* < 0.05) compared to ID; ^†^Significant difference (*P* < 0.05) compared to KV; ^§^Significant difference (*P* < 0.05) compared to AT; ^¶^Significant difference (*P* < 0.05) compared to VT

All commercial VLs showed better ease of use scores as opposed to the DL (*P* < 0.05), and the scores of the ID did not significantly differ from the DL. Notably, users repeatedly reported higher preference scores for both the VT and KV (*P* < 0.05).

## Discussion

Endotracheal intubation is a lifesaving intervention that effectively prevents aspiration and hypoxemia. Indeed, the inability to secure the airway is one of the leading causes of anesthesia-related complications [1]. Furthermore, intubation is difficult to master for novices: the initial success rate varies between 35 and 65% for intubation by medical support staff, medical students and novice anesthesia residents [15–17]. Today, VLs are used to overcome difficulties that may occur during airway management. Despite promising results, the availability of VLs in clinical practice is still considerably restricted, reportedly due to costs [6]. The VT and the ID are affordable and relatively new devices with limited but promising data reported in the literature [6, 8, 9].

Before a detailed discussion of our results, the following limitations of our study should be considered. First, all data were obtained from a monocentric mannequin study, in which interventions were accomplished by medical students. The time gap between the training and the evaluation phases of the study was 30 min; therefore, the transferability of our findings into clinical practice is questionable. Furthermore, dental trauma was assessed in a “yes” or “no” fashion, regardless of the exact number of “clicks” experienced during the attempts.

We noted high first attempt success rates (above 90%) and short ITs (less than 25 s) throughout the study, which are considered to be very good results by novices after only 15 min of training. Although, the learning process for tracheal intubation has already been studied, with a wide variety of results. With learning objective of intubation with > 90% success rate on the first attempt, previous studies found that an acceptable level of expertise was reached by 3 to 57 intubations [16, 18]. Even tough complex skills deteriorates over time, our aforementioned findings might be interesting and promising for future education programs regarding intubation by novices [19].

The LT and IT were shorter in our study than in previous reports regarding the use of DL, KV and AT, which is likely due to the short time gap between the training and evaluation sessions [2, 11, 20]. The duration of demonstration was the same as reported by Maharaj et al., although this time was 10 min longer than in the Pieters study [2, 11]. The type of the airway trainer did not differ, but training was not allowed in the aforementioned previous studies, unlike in our evaluation, which may have influenced our results [2, 11]. Cohen et al. reported that ITs were comparable to those of the Glidescope based on the ID, while we noted longer intubation times, similar to Karippacheril et al. [6, 8]. The LT and IT were shorter with commercial VLs than with the DL throughout our study, and the ID significantly underperformed in these contexts. The superiority of VLs over DL is well documented in both mannequins and in humans, which is consistent with our results [5, 21].

The use of commercial VLs significantly improved the POGO scores compared to the use of DL, but the ID proved to be similar in performance to the DL in both scenarios. This advantage of VLs has already been widely reported [22, 23]. We chose the POGO score over the Cormack and Lehane grade to express the laryngeal view due to the intra- and inter-rater reliability, which has been largely proven to be more reliable with the POGO score system [24].

The lower number of dental injuries with VLs in normal airway scenarios has already been reported. However, in Scenario B, we noted rate of dental injury using VLs that was similar to (KV, VT) or even higher than that of DL, which may be due to limited operator experience or the degree of difficulty associated with effective device insertion. This increase was more prominent with the use of the AT, possibly as a result of the bulky head due to the phone adapter. Contrary to our results, the use of the AT without the phone adapter reportedly decreases the risk of dental trauma [25].

The increase in stylet and bougie usage by the ID may be attributed to the previously discussed low POGO scores and the lack of a tube guidance channel. A tube guidance channel might improve IT by decreasing TIT, as noted in previous reports [26].

The choice between similar intubation devices in daily clinical practice is strongly based on previous experience and subjective factors. Our novice operators, based on their experience, considered commercial VLs easier to use than the DL in both scenarios, although they preferred to reuse only the KV and the VT rather than the DL. These results are consistent with those of previous studies [10, 27].

## Conclusions

In conclusion, performance with the custom-made ID in novices was at best similar, but mostly inferior, to a regular DL. Therefore, we cannot recommend the ID for inexperienced professionals in regular clinical practice until further investigations prove otherwise. Based on our results, the tested commercially available VLs can be recommended in both scenarios over the DL for students or specialists in training. Moreover, our results identify the VT as a new, promising and affordable device that is at least comparable or even superior in some aspects to the KV and AT based on the results of our scenarios.
